# Online database for mosquito (Diptera, Culicidae) occurrence records in French Guiana

**DOI:** 10.3897/zookeys.532.6176

**Published:** 2015-11-05

**Authors:** Stanislas Talaga, Jérôme Murienne, Alain Dejean, Céline Leroy

**Affiliations:** 1 CNRS; Laboratoire Ecologie des Forêts de Guyane (Ecofog; UMR 8172), Campus agronomique, 97310, Kourou, French Guiana; 2 CNRS/UPS/ENFA; Laboratoire Evolution et Diversité Biologique (EDB; UMR 5174), Université de Toulouse, 118 route de Narbonne, 31062, Toulouse, France; 3 CNRS/UPS/INP; Laboratoire Ecologie fonctionnelle et Environnement (Ecolab; UMR 5245), Université de Toulouse, 118 route de Narbonne, 31062, Toulouse, France; 4 IRD; Laboratoire de botAnique et Modélisation de l’Architecture des Plantes et des végétations (AMAP; UMR 123), Boulevard de la Lironde, TA A‐51/PS2, 34398, Montpellier, France

**Keywords:** Occurrence, French Guiana, Neotropics, mosquitoes, diversity

## Abstract

A database providing information on mosquito specimens (Arthropoda: Diptera: Culicidae) collected in French Guiana is presented. Field collections were initiated in 2013 under the auspices of the CEnter for the study of Biodiversity in Amazonia (CEBA: http://www.labexceba.fr/en/). This study is part of an ongoing process aiming to understand the distribution of mosquitoes, including vector species, across French Guiana. Occurrences are recorded after each collecting trip in a database managed by the laboratory *Evolution et Diversité Biologique*
(EDB), Toulouse, France. The dataset is updated monthly and is available online. Voucher specimens and their associated DNA are stored at the laboratory *Ecologie des Forêts de Guyane*
(Ecofog), Kourou, French Guiana. The latest version of the dataset is accessible through EDB’s Integrated Publication Toolkit at http://130.120.204.55:8080/ipt/resource.do?r=mosquitoes_of_french_guiana or through the Global Biodiversity Information Facility data portal at http://www.gbif.org/dataset/5a8aa2ad-261c-4f61-a98e-26dd752fe1c5 It can also be viewed through the Guyanensis platform at http://guyanensis.ups-tlse.fr

CEnter for the study of Biodiversity in Amazonia

*Evolution et Diversité Biologique*

*Ecologie des Forêts de Guyane*

## Introduction

Mosquitoes (Diptera: Culicidae) are probably the most medically important group of arthropods worldwide because of the ability of some species to transmit pathogens to humans (Clements 2011), causing major health issues in some parts of the world. Mosquito-borne diseases are frequent in French Guiana with malaria occurring mainly in inland areas, dengue and chikungunya in urban areas, while many lesser known crypto-arboviruses occur in sylvan and/or rural environments (Chippaux and Pajot 1983). To date, 3,543 valid species of mosquitoes have been described (Harbach 2015) and French Guiana, with 235 species, harbors one of the highest relative species densities of mosquitoes anywhere in the world (Foley et al. 2008, Talaga et al. 2015). Understanding the biology, ecology and distribution of this group is thus of primary importance.

French Guiana is mainly covered by primary rainforest and its inhabitants (*ca.* 250,000) are mostly distributed along the coast (Gond et al. 2011). While some evidence suggests that the Guiana Shield could be an early center of speciation for mosquitoes in the Neotropics (Navarro et al. 2007), the mechanisms explaining the high mosquito diversity in the region remain poorly understood.

This work is an ongoing process and should help to understand mosquito distribution across French Guiana. This database will also be used to disseminate biodiversity information related to future studies on mosquito distribution in French Guiana in general and in medical entomology and ecology in particular. We aim to promote the best practices for recording and sharing biodiversity data within our research community, and highly encourage foreign institutions to do the same. Our goal is to provide data on Guianese mosquitoes and to make available a fast and efficient tool for sharing and tracking reliable information on specimens in the form of an online database.

## Taxonomic coverage

**Description**: This database concerns all mosquito (Diptera: Culicidae) species inhabiting French Guiana. Most specimens have been identified to species level or at least to genus level. The identifications were made by the first author based most of the time on the examination of immature and adult specimens, and by using the latest taxonomic publications on the genus or on the subgenus concerned (e.g. Harbach and Peyton 2000, Motta and Lourenço-de-Oliveira 2000, Zavortink 1979). The validation of species and subspecies is based on “A Catalog of the Mosquitoes of the World (Diptera: Culicidae)” (Knight and Stone 1977) and its supplements (Knight 1978; Ward 1984, 1992; Gaffigan and Ward 1985), and the “Systematic Catalog of Culicidae” (WRBU 2015). The internal classification of the tribe Aedini is based on Wilkerson et al. (2015).

**Figure 1.**
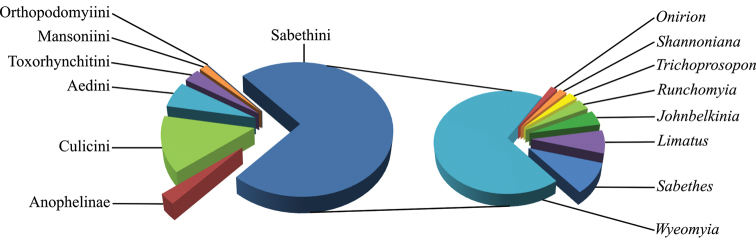
Taxonomic coverage by tribe (pie chart on the left) with a focus on the distribution of specimens by genus for the tribe Sabethini (pie chart on the right) from the dataset the “Mosquitoes of French Guiana” up to 2015. Because there are no tribes in the Anophelinae, they are represented at the subfamily level on the pie chart.

Until now, the database was mostly filled with data from studies conducted on mosquitoes breeding in phytotelmata, which explains why the Sabethini are particularly well represented in the current dataset (Fig. 1). Consequently, clades like the Anophelinae, Culicini and Mansoniini are highly underrepresented and the tribes Aedeomyiini and Uranotaeniini are not at all represented (Fig. 1). The dataset presently contains 19 genera and 81 species, including occurrences of twelve species recently recorded in French Guiana (Talaga et al. 2015), namely: *Onirion* sp. cf. Harbach & Peyton (2000), Sabethes (Peytonulus) hadrognathus Harbach, 1995, Sabethes (Peytonulus) paradoxus Harbach, 2002, Sabethes (Peytonulus) soperi Lane & Cerqueira, 1942, Sabethes (Sabethinus) idiogenes Harbach, 1994, Sabethes (Sabethes) quasicyaneus Peryassú, 1922, Runchomyia (Ctenogoeldia) magna (Theobald, 1905), Wyeomyia (Caenomyiella) sp. cf. Harbach & Peyton (1990), Wyeomyia (Dendromyia) ypsipola Dyar, 1903, Wyeomyia (Hystatomyia) lamellata (Bonne-Wepster & Bonne, 1919), Wyeomyia (Miamyia) oblita (Lutz, 1904), and Toxorhynchites (Lynchiella) guadeloupensis (Dyar & Knab, 1906).

## Taxa include

**Kingdom**: Animalia

**Phylum**: Arthropoda

**Class**: Insecta

**Order**: Diptera

**Family**: Culicidae

**Subfamilies**: Anophelinae, Culicinae.

**Tribes**: Aedeomyiini, Aedini, Culicini, Mansoniini, Orthopodomyiini, Sabethini, Toxorhynchitini, Uranotaeniini.

**Genera**: *Aedeomyia*, *Aedes*, *Anopheles*, *Chagasia*, *Coquillettidia*, *Culex*, *Deinocerites*, *Haemagogus*, *Johnbelkinia*, *Limatus*, *Lutzia*, *Mansonia*, *Onirion*, *Orthopodomyia*, *Psorophora*, *Runchomyia*, *Sabethes*, *Shannoniana*, *Toxorhynchites*, *Trichoprosopon*, *Uranotaenia*, *Wyeomyia*.

## Spatial coverage

**Figure 2.**
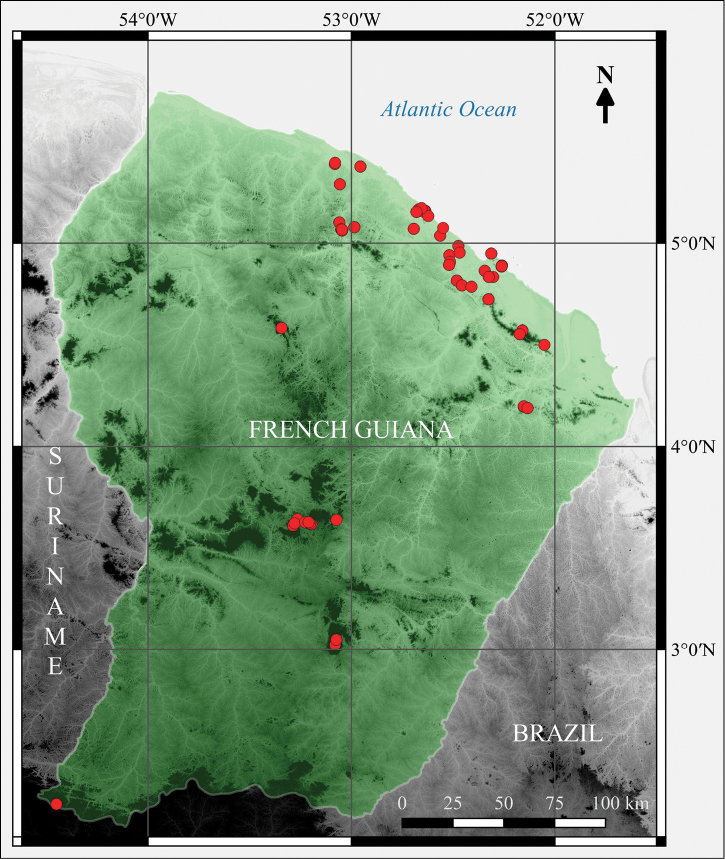
Geographical coverage of the dataset (green shade) and collecting localities (red dots) up to 2015.

**Description**: French Guiana (83,534 km²) is a French overseas region situated in South America at the eastern limit of the Guiana Shield. The latter is a mountainous tableland extending, from West to East, across Guyana, Suriname, French Guiana, as well as parts of Colombia, Venezuela and Brazil. The sampling area is delimited by the current administrative boundaries of the territory of French Guiana (Fig. 2). To the East, the Oyapock River delimits the border with Brazil. To the West, the Maroni River delimits the border with Suriname. The territory’s borders have not been constant throughout history and a large portion of northern Brazil was disputed between France and Brazil during the 19th century. As a result, the type locality of Counani, French Guiana where the *nomen dubium Culex americanus* Neveu-Lemaire, 1902 was described (Belkin et al. 1971) is currently in Brazil. Even though French Guiana is a French overseas region, all occurrences have been recorded under the country “French Guiana” to comply with the ISO 3166-1 standard.

**Geographical methods**: GPS coordinates were obtained using a Garmin GPSmap 60CSx device or higher equivalent of the GPSmap series. The World Geodetic System 1984 (WGS 84) was used as geodetic system and associated with UTM 21-22 N for map projection.

**Coordinates**: 2°5'24"N and 5°50'60"N Latitude; 54°36'36"W and 51°31'48"W Longitude

## Temporal coverage

**Notes**: From October 2013 to present.

## Project description

**Title**: Mosquitoes of French Guiana

**Personnel**: Stanislas Talaga

**Study area descriptions**: Collecting trips were conducted in various locations throughout French Guiana ranging from urban to pristine environments.

**Design description**: This database was originally built from studies on mosquito-phytotelm associations at the scale of French Guiana. Immature mosquitoes were collected from at least 30 water-holding structures per phytotelm species, per locality. However, the extent of the sampling area was not standardized between the different localities. The database also contains some records of opportunistically sampled immature and adult mosquitoes conducted by the first author.

**Funding**: Data for this resource have been obtained within the framework of the projects BIOHOPSYS and DIADEMA from the CEBA (CEnter for the study of Biodiversity in Amazonia) and thanks to a PhD fellowship from the *Université Antilles-Guyane* awarded to Stanislas Talaga. CEBA is funded by an *Investissement d’Avenir* grant managed by the French *Agence Nationale de la Recherche*
(ANR) under grant number ANR-10-LABX-25-CEBA.

## Methods

**Study extent description**: Study sites were located throughout French Guiana.

**Sampling description**: The following techniques were used; however, not all techniques were used at every collecting site and the sampling design may not have been the same at all sites.

Immature container mosquitoes were collected by extracting plant-held water using a great variety of sucking devices in order to fit the great variety of plant structures and water volumes. On some occasions, natural and artificial ovitraps were used, including bamboo stumps, CDC ovitraps and artificial bromeliads installed at ground or canopy level. Immature mosquitoes from larger bodies of water were collected by using a kick net. Adult mosquitoes were attracted in the field by human bait and captured with a butterfly net or with an entomological aspirator when they alighted.

**Processing**: Whenever possible, samples were brought back alive to the laboratory. Immature mosquitoes were individually reared in 2 mL Eppendorf tubes and placed in a climatic chamber at 28 °C to obtain adults. When a sufficient number of adults was obtained, some of them were stored in individual tubes containing 95% ethanol. Fourth instar and pupal skins were also sorted and stored in individual tubes containing 70% ethanol. Laboratory-reared adults and adults issued from field capture were killed by freezing. Three legs from the right side of each specimen were then carefully dissected and kept in a separate vial containing 95% ethanol and stored at -20 °C for further molecular investigations. Adults were mounted on their right side on a pin point attached to a No. 3 stainless steel insect pin and stored in entomological boxes. Specimen codes are based on the name of the collection followed by a unique serial number as proposed by Gaffigan and Pecor (1997). The same code was used for all of the biological material issued from the same specimen. When it was impossible to bring live samples back to the laboratory and rearing was not possible either, specimens were stored directly in 95% ethanol in the field.

**Figure 3.**
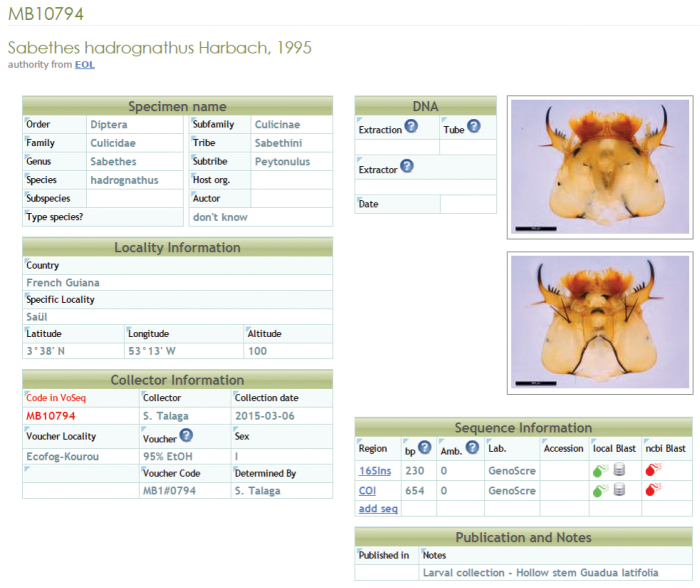
Sample data entry of our online database (http://mosquitoes.ups-tlse.fr with restricted access) holding the “Mosquitoes of French Guiana” dataset.

Selected specimens were photographed using a Leica DFC450 camera mounted on a Leica MZ16 macroscope under a light dome simulating natural light. Images were Z-stacked using the Leica LAS Z-stacking module. Montage pictures and collecting information for each specimen are stored in an online Voseq database (Peña and Malm 2012) managed by the EDB laboratory (Fig. 3) and viewable through the Guyanensis GIS web platform at http://guyanensis.ups-tlse.fr, through the Global Biodiversity Information Facility (GBIF) at http://www.gbif.org/dataset/5a8aa2ad-261c-4f61-a98e-26dd752fe1c5 or alternatively through the local Integrated Publishing Toolkit (IPT) at http://130.120.204.55:8080/ipt/resource.do?r=mosquitoes_of_french_guiana

Specimens are initially curated at the Ecofog laboratory by Stanislas Talaga and can be deposited in museums for further taxonomic study.

**Quality control description**: Considering different sources of GPS errors (such as ionosphere delay and signal multipath), we estimate the accuracy of the coordinates to be around 30 meters at a 95% confidence level.

## Data resources

**Dataset title**: Mosquitoes of French Guiana

**Resource**: r=mosquitoes_of_french_guiana

**Character encoding**: UTF-8

**Format name**: Darwin Core Archive (Darwin Core Task Group 2009)

**Format version**: 1.0

**Distribution**: http://130.120.204.55:8080/ipt/resource.do?r=mosquitoes_of_french_guiana

**Publication date of data**: 2015-06-12

**Language of database**: English

**License of use**: Other

**Date of metadata creation**: 2014-12-10

**Hierarchy level**: Dataset

## Usage rights

**IP rights notes**: This work is licensed under a Creative Commons Attribution- NonCommercial 4.0 International Public License. http://creativecommons.org/licenses/by-nc/4.0/ Users of this resource should also comply with the CEBA data sharing agreement available here: http://www.labex-ceba.fr/assets/CEBA_Data_Sharing_Agreement_nov2013.pdf
